# Awareness and Preparedness of Field Epidemiology Training Program Graduates to Respond to COVID-19 in the Eastern Mediterranean Region: Cross-Sectional Study

**DOI:** 10.2196/19047

**Published:** 2020-09-18

**Authors:** Mohannad Al Nsour, Yousef Khader, Abdulwahed Al Serouri, Haitham Bashier, Shahd Osman

**Affiliations:** 1 Global Health Development/Eastern Mediterranean Public Health Network Amman Jordan; 2 Jordan University of Science and Technology Irbid Jordan; 3 Yemen Field Epidemiology Training Program Sana’a Yemen; 4 Sudan Field Epidemiology Training Program Khartoum Sudan

**Keywords:** COVID-19, infection, preparedness, awareness, Jordan, Yemen, Sudan

## Abstract

**Background:**

The Field Epidemiology Training Program (FETP) is a 2-year training program in applied epidemiology. FETP graduates have contributed significantly to improvements in surveillance systems, control of infectious diseases, and outbreak investigations in the Eastern Mediterranean Region (EMR).

**Objective:**

Considering the instrumental roles of FETP graduates during the coronavirus disease (COVID-19) crisis, this study aimed to assess their awareness and preparedness to respond to the COVID-19 pandemic in three EMR countries.

**Methods:**

An online survey was sent to FETP graduates in the EMR in March 2020. The FETP graduates were contacted by email and requested to fill out an online survey. Sufficient number of responses were received from only three countries—Jordan, Sudan, and Yemen. A few responses were received from other countries, and therefore, they were excluded from the analysis. The questionnaire comprised a series of questions pertaining to sociodemographic characteristics, knowledge of the epidemiology of COVID-19, and preparedness to respond to COVID-19.

**Results:**

This study included a total of 57 FETP graduates (20 from Jordan, 13 from Sudan, and 24 from Yemen). A total of 31 (54%) graduates had attended training on COVID-19, 29 (51%) were members of a rapid response team against COVID-19, and 54 (95%) had previous experience in response to disease outbreaks or health emergencies. The vast majority were aware of the main symptoms, mode of transmission, high-risk groups, and how to use personal protective equipment. A total of 46 (81%) respondents considered themselves well prepared for the COVID-19 outbreak, and 40 (70%) reported that they currently have a role in supporting the country’s efforts in the management of COVID-19 outbreak.

**Conclusions:**

The FETP graduates in Jordan, Sudan, and Yemen were fully aware of the epidemiology of COVID-19 and the safety measures required, and they are well positioned to investigate and respond to the COVID-19 pandemic. Therefore, they should be properly and efficiently utilized by the Ministries of Health to investigate and respond to the current COVID-19 crisis where the needs are vastly growing and access to outside experts is becoming limited.

## Introduction

In 2014, the Global Health Security Agenda was launched to accelerate progress toward implementation of the International Health Regulations (IHR) 2005, so that all countries are able to rapidly detect, respond to, and control public health emergencies [1,2]. This emphasized the role of Field Epidemiology Training Programs (FETP) to ensure global health security [3]. FETP is originally a 2-year training program in applied epidemiology that is established to produce well-trained multidisciplinary public health professionals who are competent in health surveillance systems, outbreak detection and response to health threats, and management of emerging and re-emerging diseases [4,5]. In recent years, the Ministries of Health in some countries have recognized the importance of strengthening the capacity of the public health workforce at all levels of the public health system. In response, a three-tiered “pyramid” model of training (ie, advanced: 2 years, intermediate: 9 months, and basic: 3 months) was adopted. However, only few countries had achieved the Joint External Evaluation target of having 1 trained field epidemiologist (or equivalent) per 200,000 people [6].

The current coronavirus disease (COVID-19) and the previous outbreak of severe acute respiratory syndrome raised concerns about the continued global vulnerability to infectious disease threats and the poor preparedness to respond to such threats [2,7]. This vulnerability underscores the need for field epidemiology workforce and capacity in all countries of the world at all levels of the health care and public health system.

The Eastern Mediterranean Public Health Network (EMPHNET) has helped to launch, establish, and support several FETPs in many countries in the Eastern Mediterranean Region (EMR). As service-based training programs implementing competency-based training under the supervision of qualified mentors/supervisors, FETPs are focused on building workforce capacity in public health surveillance, outbreak investigations, epidemiological methods, laboratory and biosafety, risk communications, health-related surveys, and evaluation of the impact of prevention and control programs. The programs are established within the Ministries of Health and have access to technical assistance from the Centers for Disease Control and Prevention.

In the EMR, FETP residents and graduates have contributed significantly to improvements in surveillance systems, control of infectious diseases, and outbreak investigations [8] and have been instrumental in controlling many past epidemics including Middle East respiratory syndrome (MERS) [9,10] and dengue fever outbreak [11]. During the current emergency, the FETP graduates played a key role in actions responding to COVID-19 including developing preparedness plans, supporting and evaluating the surveillance system to identify the gaps and needs, assessing the needs in health facilities for isolation rooms, case investigations, points of entry/arrivals screening and follow-up, quarantine and isolation protocols, transferring cases, risk communication, and training on infection control. FETP graduates in many EMR countries are currently members of different technical, advisory, and coordination committees that manage the COVID-19 threats in the region. Moreover, they are involved in developing/adapting local guidelines, protocols, and case definitions for health professionals to implement various interventions. Considering their instrumental roles during the COVID-19 crisis, this study aimed to assess the awareness and preparedness of FETP graduates in three EMR countries to respond to the COVID-19 pandemic.

## Methods

### Study Population

The study population consisted of advanced FETP graduates in three countries—Jordan, Sudan, and Yemen. An online questionnaire was sent to FETP graduates in the EMR in March 2020. The email addresses of the graduates were extracted from the FETP database at EMPHNET. The FETP database includes contact information and identifying information on the FETP residents and graduates in the region. The FETP graduates were contacted by email and requested to fill an online survey. The purpose of the study was explained to all contacted persons; they were informed that their participation is voluntary and were assured of confidentiality and privacy. Ethical approval was obtained from the Institutional Review Board at Jordan University of Science and Technology. A sufficient number of responses were received from only three countries—Jordan, Sudan, and Yemen. Few responses were received from other countries, and therefore, they were excluded from the analysis.

### Study Questionnaire

An online questionnaire was developed using PollDaddy (Automaticc Inc) to collect the data. The questionnaire was anonymous to maintain the privacy and confidentiality of all information collected in the study. Questions of the survey were developed after reviewing pertinent literature and the international guidelines. The questionnaire was designed in English and comprised a series of questions pertaining to sociodemographic characteristics; knowledge of FETP graduates about the epidemiology of COVID-19; and their attitude, preparedness, and perception of COVID-19. The respondents were requested to answer questions on incubation period, symptoms of the disease, mode of transmission of the COVID-19, infection control measures for preventing COVID-19, high-risk groups, and diagnostic tests. Other questions were added to assess their preparedness to respond to COVID-19. The questionnaire was pilot tested on 10 FETP graduates in Jordan.

### Data Analysis

Data were analyzed using IBM SPSS version 24 (IBM Corp). Descriptive statistical analysis was used to describe items included in the survey. Means and standard deviations were used to describe the continuous variables, and percentages were used to describe the categorical data.

## Results

### Participant Characteristics

This study included a total of 57 FETP graduates (20 from Jordan, 13 from Sudan, and 24 from Yemen) from the three studied countries. Table 1 shows the characteristics of the respondents. Almost three-quarters (n=40, 70%) of the participants were male, 30 (53%) were aged <40 years (mean 39.2, SD 8.1 years), and 24 (42%) had ≥10 years of work experience (mean 10.2, SD 8.2 years). Of all participants, 46 (80.7%) were employed by the Ministry of Health. A total of 31 (54%) graduates had attended training on COVID-19, 29 (51%) were members of a rapid response team against COVID-19, and 54 (95%) had previous experience in response to disease outbreaks or health emergencies. The FETP graduates in Jordan were more likely to be in the response team against COVID-19 than their counterparts in Sudan and Yemen.

**Table 1:** The characteristics of 57 Field Epidemiology Training Program graduates in Jordan, Sudan, and Yemen.

Variable			Country						
			Jordan (n=20), n (%)		Sudan (n=13), n (%)		Yemen (n=24), n (%)		Total (N=57), n (%)
**Gender**									
	Female	5 (25)		8 (62)		4 (17)		17 (30)	
	Male	15 (75)		5 (38)		20 (83)		40 (70)	
**Age (years)**									
	<40	14 (70)		9 (69)		7 (29)		30 (53)	
	≥40	6 (30)		4 (31)		17 (71)		27 (47)	
**Work experience (years)**									
	<10	14 (70)		7 (54)		11 (46)		32 (56)	
	≥10	5 (25)		6 (46)		13 (54)		24 (42)	
Attended training on COVID-19^a^			15 (75)		6 (46)		10 (42)		31 (54)
A member of a rapid response team against COVID 19			17 (85)		7 (54)		5 (21)		29 (51)
Previous experience in response to disease outbreaks or health emergencies			18 (90)		13 (100)		23 (96)		54 (95)

^a^COVID-19: coronavirus disease.

### Awareness of the Epidemiology of COVID-19 Infection

Table 2 shows the FETP graduates’ awareness of the epidemiology of COVID-19 infection. All respondents were aware that the incubation period is between 1 and 14 days and that the main symptoms of the COVID-19 infection include fever and cough. The majority (n=56, 98%) reported shortness of breath, 44 (77%) reported sore throat, 38 (67%) reported runny nose, and 38 (67%) reported that COVID-19 may present with no symptoms. All were aware that the mode of transmission of COVID-19 includes coughing and sneezing, and the majority reported knowledge of transmission through hand shaking (n=50, 88%) and touching surfaces such as doorknobs and tables (n=51, 89%). The majority (n=56, 98%) reported that real-time polymerase chain reaction (PCR) with respiratory material is the diagnostic test for COVID-19. When they were asked about what should be considered to identify patients at risk of COVID-19, 55 (96%) reported history of travel to areas with transmission of COVID-19, 51 (89%) reported history of contact with possibly infected patients, 46 (81%) reported the presence of symptoms of a respiratory infection, and 2 (4%) reported the presence of symptoms of diarrhea. The majority were aware of high-risk groups such as people with immune system deficiency (n=55, 96%), people with chronic diseases (n=55, 96%), and health care providers (n=52, 91%).

**Table 2:** The Field Epidemiology Training Program graduates’ awareness of the epidemiology of COVID-19 infection.

Variable		Country				
		Jordan (n=20), n (%)	Sudan (n=13), n (%)	Yemen (n=24), n (%)	Total (N=57), n (%)	
**Symptoms of the COVID-19^a^ infection**						
	Fever	20 (100)	13 (100)	24 (100)		57 (100)
	Cough	20 (100)	13 (100)	24 (100)		57 (100)
	Shortness of breath	20 (100)	12 (92)	24 (100)		56 (98)
	Sore throat	19 (95)	6 (46)	19 (79)		44 (77)
	Runny nose	13 (65)	6 (46)	19 (79)		38 (67)
	None	13 (65)	8 (62)	17 (71)		38 (67)
	Joint/muscle pain	10 (50)	8 (62)	14 (58)		32 (56)
	Diarrhea	12 (60)	2 (15)	10 (42)		24 (42)
	Red eyes	2 (10)	2 (15)	4 (17)		8 (14)
	Rash	0 (0)	2 (15)	0 (0)		2 (4)
**Mode of transmission**						
	Coughing and sneezing	20 (100)	13 (100)	24 (100)		57 (100)
	Hand shaking	16 (80)	12 (92)	22 (92)		50 (88)
	Touching surfaces	16 (80)	13 (100)	22 (92)		51 (89)
Diagnostic test: real-time polymerase chain reaction with respiratory material		20 (100)	13 (100)	23 (96)	56 (98)	
**Criteria to identify patients at risk of COVID-19**						
	History of travel to areas experiencing transmission of COVID-19	20 (100)	13 (100)	22 (92)		55 (96)
	History of contact with possible infected patients	17	13 (100)	21 (88)		51 (89)
	Respiratory infection symptoms	16	13	17 (71)		46 (81)
	Diarrhea symptoms	0 (0)	1 (8)	1 (4)		2 (4)

^a^COVID-19: coronavirus disease.

### Awareness of Safety Measures and Preparedness to Respond to COVID-19

Table 3 shows the FETP graduates’ awareness of safety measures and their preparedness to respond to COVID-19. All FETP graduates reported that they know how to use personal protective equipment and 50 (88%) reported that they know how to perform isolation procedures to minimize chances for exposure. More than half (n=33, 58%) reported that they are highly confident to handle suspected COVID-19 patients. All reported that they do not mind working in a place where patients with COVID-19 are treated. The majority (n=53, 93%) reported that they are up to date on safety measures for COVID-19. A total of 46 (81%) respondents considered themselves well prepared for the COVID-19 outbreak and 40 (70%) reported that they currently have a role in supporting the country efforts in the management of COVID-19 outbreak. Almost half (total: n=26, 46% [Jordan: 12, 60%; Sudan: 3, 23%; Yemen: 11, 46%]) reported that they think that their countries are prepared for the management of the COVID-19. However, only 11 of 26 persons (42%) reported that they are satisfied with the preparedness of their countries to respond to the COVID-19 pandemic. All reported that they know whom to contact in a situation where there has been an unprotected exposure to a known or suspected COVID-19 patient.

**Table 3:** The Field Epidemiology Training Program graduates’ awareness of safety measures and their preparedness to respond to COVID-19.

Variable			Country					
			Jordan (n=20), n (%)		Sudan (n=13), n (%)		Yemen (n=24), n (%)	Total (N=57), n (%)
Know how to use personal protective equipment			20 (100)		13 (100)		24 (100)	57 (100)
Know how to perform isolation procedures			17 (85)		13 (100)		20 (83)	50 (88)
**Level of confidence in handling suspected COVID-19^a^ patients**								
	High	8 (40)		9 (69)		17 (71)		34 (60)
	Low	12 (60)		1 (8)		7 (29)		20 (35)
	Not confident	0 (0)		3 (23)		0 (0)		3 (5)
Have the contact of the International Health Regulations focal point in the country			14 (70)		9 (69)		16 (67)	39 (68)
Up to date on safety measures for COVID-19			18 (90)		11 (85)		24 (100)	53 (93)
Do not mind dealing with and handling patients with COVID-19			20 (100)		12 (92)		24 (100)	56 (98)

^a^COVID-19: coronavirus disease.

### Perception of COVID-19

The majority (n=53, 93%) perceived COVID-19 as moderately dangerous to very dangerous, 30 (53%) reported that it is more dangerous than severe acute respiratory syndrome, and 33 (58%) reported that it is more dangerous than MERS-CoV (coronavirus). About one-tenth (n=7, 12.3%) believed that COVID-19 is not currently a serious public health issue. A total of 31 (54%) respondents were aware of that COVID-19 symptoms often resolve with time.

### Sources of Information About COVID-19

The majority of FETP graduates reported multiple sources for the information they receive about COVID-19 including the Ministry of Health (n=53, 93%), television and radio (n=32, 56%), Epishares (n=32, 56%), and social media (n=32, 56%).

## Discussion

This survey provides insight on the preparedness of FETP graduates from three EMR countries and their level of awareness of COVID-19 epidemiology at the time of the COVID-19 pandemic. The three programs had different durations since their establishment. The FETP graduates in Jordan were more likely to be in the response team against COVID-19 than their counterparts in Sudan and Yemen. This might be explained by the fact that both Sudan and Yemen were not reporting cases at the time of data collection. However, the FETP graduates were involved in preparedness activities for COVID-19. Males were predominant in the sample, which can be explained by the higher percentages of males who were enrolled in these program.

To conduct and respond to an infectious disease outbreak such as COVID-19, the FETP graduates should be aware of the basics of infectious disease including agents and hosts, mode of transmission, signs and symptoms, and control measures [12].

Knowing the COVID-19 incubation period is crucial for FETP graduates to protect themselves from the subclinical infection [13]. All respondents identified the correct incubation period of 1-14 days [14]. Almost all respondents were able to identify cough and fever as the main symptoms of COVID-19 [15].

Although the majority of respondents perceived COVID-19 as a dangerous infection, all reported that they have no problem with handling patients with COVID-19. This reflects the positive attitudes of FETP graduates toward patients with COVID-19 and their willingness to control the pandemic. This is not surprising because FETP residents and graduates are trained to conduct outbreak investigation and respond to public health threats. Moreover, this reflects their high level of confidence in dealing with patients with COVID-19 because all were aware of how to use personal protective equipment and perform isolation procedures on patients to minimize chances for exposure.

This study showed that the majority of respondents receive information about COVID-19 from the Ministries of Health. Reliance on the Ministry of Health data and reports reflects that the information they gain is credible. Therefore, Ministries of Health need to make sure that all essential information and educational materials are posted on the ministries’ website during the outbreak.

Half of the respondents gained information on COVID-19 from television and radio, Epishares, and social media. It is worth mentioning the EpiShares was identified as a source of information by many of the FETP graduates. Epishares is a networking platform powered by Global Health Development/EMPHNET [16]. The Epishares platform offers a space for public health professionals from the region and beyond to exchange ideas, discuss issues, share experiences, and share documents of interest. It functions like other social media channels, thus offering the ability to create pages and groups as well as share posts videos, photos, polls, documents, and more. Throughout the COVID-19 pandemic, the platform was used as a hub for news and knowledge and to provide regular updates from credible news sources about the pandemic. These updates are shared via a page on this platform dedicated to this purpose and titled “COVID-19 Updates.” Furthermore, the platform holds a group dedicated to FETP. The group serves as a private space for FETP directors, advisors, and support teams to share guidelines, reports, practices, and exchange ideas in the fight against this pandemic.

### Conclusions

The FETP graduates in Jordan, Sudan, and Yemen were fully aware of the epidemiology of COVID-19 and the safety measures. In addition, they are well positioned to investigate and respond to COVID-19 pandemic. Therefore, they should be properly and efficiently utilized by the Ministries of Health to investigate and respond to the current COVID-19 crisis where the needs are vastly growing and access to outside experts is becoming limited. Moreover, the current pandemic revealed the increased demand for more FETP graduates, and thus, there is a need to maintain and continue to improve the quality and reach of FETPs by expanding the number of countries with access to these programs and expanding the FETP tiered training approach (advanced, intermediate, and basic), especially in countries with similar health workforce challenges.

## Abbreviations

COVID-19: coronavirus disease
EMPHNET: Eastern Mediterranean Public Health Network
FETP: Field Epidemiology Training Program
IHR: International Health Regulations
MERS: Middle East respiratory syndrome
PCR: polymerase chain reaction
